# Notice to the Members of the U. S. V. M. A.

**Published:** 1896-08

**Authors:** 


### SOCIETY PROCEEDINGS.

### NOTICE TO THE MEMBERS OF THE U. S. V. M. A.

THE local committee of arrangements for the Buffalo meeting, September 1st, 2d, and 3d next, are desirous that all members, delegates and other veterinarians who anticipate attending the convention will give notice of the same to H. D. Martin, V.S., Secretary Local Committee, 481 Rhode Island St., Buffalo, N. Y. Dr. Edward McLeod, President.

Dr. H. D. Gill will prepare a paper for the meeting at Buffalo on the "Relation of Veterinary Colleges and Veterinarians to Horseshoers."

The New York County Society will send delegates to the Buffalo meeting.

President Hoskins, of the U. S. V. M. A., has recently been accorded honorary membership in the New York County Veterinary Medical Association, and their honorary certificate of membership was conferred upon him.

President Ridge, of the Pennsylvania State Veterinary Medical Association, will be among the list of applicants for membership at Buffalo.

The mutual recognition of students by colleges belonging to the Association of Veterinary Faculties will be discussed by Prof. H. D. Gill at the meeting of the college faculties at Buffalo.

Major Edwin Jewett, of Buffalo, will welcome the visiting members and delegates to the "Queen City of the Lakes."

Owing to the prospects of an unusually large outpouring of the profession the sessions of the convention will be held in the lecture-room of the Buffalo University. This change from the Colonial parlor of the Genesee Hotel has been made because of the large accommodations afforded at the University.

Secretary Morton has kindly consented to give the largest latitude possible in the way of leaves of absence to those connected with the Bureau of Animal Industry consistent with the necessities of the service. An invitation has been extended Secretary Morton to be with us, and is now under consideration at his hands.

Dr. Merillat is out with an important letter of inquiry among the veterinarians, and everyone should afford him the information desired. His topic at Buffalo will provoke an interesting discussion, and there will be great variance of opinion on the subject.

There is one officer who will more than merit a reëlection. Secretary Stewart has been assiduous in his work, and he will present a better state of affairs than has been our lot for three years. He should receive a unanimous recall to this place of so much importance.

Dr. Frank H. Miller, of Burlington, Vermont, now at Berlin, Germany, will present a paper at the coming meeting at Buffalo on "Diabetes Mellitus in Dogs."

President Hoskins has finally secured the consent of Ex-President Williams to prepare a paper for the Buffalo meeting. His subject, "Physiological Variations," is one of great interest, and the members will be assured of a rich treat in store, for the writer has been one of the best contributors in recent years to the Association's original work.

Chairman Harger, of the Committee on Diseases, is out with a letter of inquiry among the members and profession which should merit a prompt reply from all those who receive it, whereby they may be mutually afforded a general knowledge of the prevalence of the several diseases enumerated among the live stock of our country. This is an important work and every effort should be made to make it as complete as possible.

The Committee on Intelligence and Education will consider in their report the following leading points: The curriculum required in the different American schools; the curriculum required in other countries; the most conservative method of procedure to obtain a uniform standard of matriculation and curriculum for the American schools. An appeal to the advanced-standard schools to use great caution in the selection of their teaching-staffs.

State Secretaries should be zealously looking over their respective States with a view of drawing into the national organization the strongest and best members of the profession.

Dr. Hinebauch will continue his interesting reports on "Feeding Millet," a subject of much interest, importance, and with which he has had much experience and given deep and intelligent study.

Veterinarian A. T. Peters is doing valuable missionary work among the veterinarians connected with Agricultural Experiment Stations in the interest of our Buffalo meeting. The advisability of forming an organization of these veterinarians to hold meetings in connection with the annual gathering of the U. S. V. M. A. is being agitated.

Veterinarians attending either the Ohio or New York State meetings in conjunction with the national convention will have the advantage of the reduced railroad rates by attendance upon one day of the latter's sessions.

The Missouri Valley Veterinary Medical Association passed by a unanimous vote a resolution to invite the U. S. V. M. A. to hold the meeting for 1897 at Kansas City, and instructed its officers to secure the meeting if possible.

Dr. Joseph Hughes, of Chicago, will be among those from the Windy City to participate in the deliberations at Buffalo.

Secretary Stewart and Chairman Williams, of the Publication Committee, say it wholly lies with the members as to whether we have a compilation of our proceedings at Buffalo or not. Pay up your arrearages, fellow-members, and make sure this much-to-be-desired part of our history.

Who will be our next President?

Chicago will send many of her strongest devotees of the profession.

The leading cities of the country will be better represented at Buffalo than at any of our meetings for years. And why not?

Chairman Turner, of the Army Legislation Committee, is sanguine of successful legislation by the next Congress.

Dr. W. H. Dalrymple, of Baton Rouge, Louisiana, will read at Buffalo a paper entitled "Southern Veterinary Experience."

Dr. Jas. A. Waugh, of Pittsburg, Pa., will introduce the subject of "Toe-clip Injuries," with a paper and report of a number of cases.

The leading subjects for consideration at Buffalo will be assigned speakers who will lead the discussion of the same.

Much complaint and criticism may be heard around of illegitimate methods of obtaining practice among members of the Association. These charges can only be acted upon by those injured making them in writing, and this should be done in the interest and for the honor of the Association and that justice may be meted out to all.

Chairman Harger reports a large number of replies received to his letter of inquiry bearing upon the prevalence and location of the contagious and infectious diseases prevailing in our country during the past year.

Among the leading topics under which the subject of tuberculosis will be considered are: First, should indemnity be paid for cattle that show clinical symptoms of tuberculosis? If not, how best may traffic in such animals be prevented? Second, if it is granted, as it seems that it must be, that tuberculosis is spread largely through the milk from tuberculous animals; but that it is not so spread by the use of the cooked flesh of such animals as food; if, therefore, it is desired to purify the milk-supply by removing all of the tuberculous animals from the herds supplying a district with milk, what shall be done with the tuberculous cattle so removed? If it is decided that their flesh may be safely used, cooked, as food, is there any governmental restriction that should be placed upon its preparation or sale? If so, should the flesh of all tuberculous animals be rendered or otherwise destroyed? If it is considered that a portion of this flesh is fit for food, can the Association lay down any practical rules at which the line between the safe and unsafe beef and veal may be clearly defined to butchers and inspectors, neither of whom are as a rule professional men? The necessity of systematic examination of cattle as it is being conducted in Massachusetts. The after-effects of tuberculin, or its influence on healthy cattle. The accuracy of tuberculin. The Danish system of controlling tuberculosis. The desirability of examining with tuberculin all animals that enter interstate trade. The virulence and dangers of tuberculous milk or milk from tuberculous cattle.

